# Genome-Wide Analysis of Whole Human Glycoside Hydrolases by Data-Driven Analysis in Silico

**DOI:** 10.3390/ijms20246290

**Published:** 2019-12-13

**Authors:** Takahiro Nakamura, Muhamad Fahmi, Jun Tanaka, Kaito Seki, Yukihiro Kubota, Masahiro Ito

**Affiliations:** 1 Advanced Life Sciences Program, Graduate School of Life Sciences, Ritsumeikan University, 1-1-1 Nojihigashi, Kusatsu, Shiga 525-8577, Japan; taka080hiro@gmail.com (T.N.); gr0343rp@ed.ritsumei.ac.jp (M.F.); tj01732@gmail.com (J.T.); sj0036kf@ed.ritsumei.ac.jp (K.S.); 2 Department of Bioinformatics, College of Life Sciences, Ritsumeikan University, 1-1-1 Nojihigashi, Kusatsu, Shiga 525-8577, Japan; yukubota@fc.ritsumei.ac.jp

**Keywords:** glycoside hydrolase, glycan, phylogenetic profiling

## Abstract

Glycans are involved in various metabolic processes via the functions of glycosyltransferases and glycoside hydrolases. Analysing the evolution of these enzymes is essential for improving the understanding of glycan metabolism and function. Based on our previous study of glycosyltransferases, we performed a genome-wide analysis of whole human glycoside hydrolases using the UniProt, BRENDA, CAZy and KEGG databases. Using cluster analysis, 319 human glycoside hydrolases were classified into four clusters based on their similarity to enzymes conserved in chordates or metazoans (Class 1), metazoans (Class 2), metazoans and plants (Class 3) and eukaryotes (Class 4). The eukaryote and metazoan clusters included *N*- and *O*-glycoside hydrolases, respectively. The significant abundance of disordered regions within the most conserved cluster indicated a role for disordered regions in the evolution of glycoside hydrolases. These results suggest that the biological diversity of multicellular organisms is related to the acquisition of *N*- and *O*-linked glycans.

## 1. Introduction

Glycans are present in various biological molecules including glycoproteins, glycolipids and proteoglycans and in more than half of all human proteins. Glycans are widely distributed in eukaryotes, bacteria and archaea [1] and have similar structures in different organisms, including yeasts, plants, insects and chordates [2]. The high conservation of glycans in different species is biologically meaningful [3]. Human glycans can be classified into four major categories: *O*-linked (mucin-type) glycans, *N*-linked glycans, glycosphingolipids, and glycosaminoglycans. These glycans play important roles in vivo, including in cell membrane/extracellular matrix (ECM) construction, cell adhesion, protein stabilisation and transmission of information [4,5]. Abnormalities in glycan structures are closely related to certain diseases such as neurological disorders, cancer metastasis, Alzheimer’s disease and diabetes [6]. The diversity of glycan functions depends on the diversity of glycan structures, i.e., the combination of monosaccharides constituting a glycan, differences in binding sites and differences in branching modes. However, the mechanisms mediating the acquisition of various glycan categories, balance between biosynthesis and degradation, and essential biological significance of glycans are unclear.

The biosynthesis and degradation of various glycan structures are mainly catalysed by glycosyltransferases and glycoside hydrolases, respectively. Glycosyltransferases function to regulate the elongation of glycans, and variations in glycosyltransferases result in diverse substrate specificities such as the type of sugar to be transferred and specific binding position of the sugar. For genome-wide evolutionary analysis of glycosyltransferases, we previously performed lineage profile analysis of 173 human glycosyltransferases [3]. The results indicate that human glycosyltransferases can be roughly divided into four categories based on their similarity to enzymes conserved in deuterostomes (Class 1), metazoans (Class 2), eukaryotes (Class 3), and eukaryotes, bacteria and archaea (Class 4). Two glycosyltransferase groups, synthesise *O*- and *N*-linked glycans, are present in the Golgi apparatus in deuterostomes and metazoans and in the endoplasmic reticulum of eukaryotes. Thus, we found that the localisation and function of glycosyltransferases conserved among deuterostomes, metazoans and other eukaryotes were distinctly different. Furthermore, our findings suggested that *N*-linked glycan structures existed before *O*-linked glycans during the evolution of these molecules in humans [3].

Glycoside hydrolases have substrate specificities similar to those of glycosyltransferases; however, many of glycosyltransferases have a strict substrate specificity, whereas glycoside hydrolases show a looser substrate specificity. Glycoside hydrolases function to cleave glycosidic bonds in glycans, and many are in lysosomes [7]. In addition to glycan degradation in lysosomes, glycoside hydrolases are closely associated with in vivo functions, such as the quality control of proteins by the processing of high-mannose-type (*N*-linked-type) glycans and remodelling of ECM comprising *O*-linked glycans and glycosaminoglycans [8]. Notably, glycoside hydrolases have been shown to play roles in lysosomal storage diseases. The lysosome is an intracellular organelle that decomposes waste products via the functions of various hydrolytic enzymes. Lysosomal storage diseases are caused by the accumulation of undegraded substances because of genetic abnormalities affecting the expression of glycoside hydrolases in lysosomes [9]. Symptoms of lysosomal storage diseases are diverse and severe. Additionally, both the synthesis of glycans and decomposition of glycans are involved in biological functions; however, the detailed functions of sugar hydrolases in lysosomes have not been determined. Particularly, the roles of glycoside hydrolases for *O*-linked glycans are unclear in lysosomal storage diseases [8].

Protein evolution is driven by function, which critically depends on the structure. This is supported by comparison of evolutionary rates between ordered and disordered structured proteins. Disordered regions commonly evolve faster than ordered structures [10,11,12,13,14] because of differences in the relative constraints that maintain folding interactions [15]. However, there are exceptions to this rule. For instance, specific functional binding and modification regions of a disordered structure are constrained [13,14,16], thus introducing heterogeneity into evolutionary rates.

In this study, we evaluated glycan degradation by performing a genome-wide analysis of 319 human glycoside hydrolases. By comparing the results of analysis of glycosyltransferases [3] and their protein structures, we clarified the acquisition process of each glycan category during evolution.

## 2. Results

### 2.1. Human Glycoside Hydrolase Dataset

In this study, 319 human glycoside hydrolases (Table S2) were retrieved from the UniProt [17], CAZy [18], and BRENDA [19] databases. The dataset was verified using the Gene Ontology (GO) term GO:0016798. Of the 319 human glycoside hydrolases in the dataset, 251 overlapped with glycoside hydrolases in the GO database (Table S1); among the 251 genes involved in the GO, 178 genes overlapped with glycoside hydrolases in the InterPro database [20]. Most data extracted using GO were related to nucleic acid-related glycoside hydrolases.

### 2.2. Human Glycoside Hydrolases Belong to Four Evolutionary Classes

**Figure 1:**
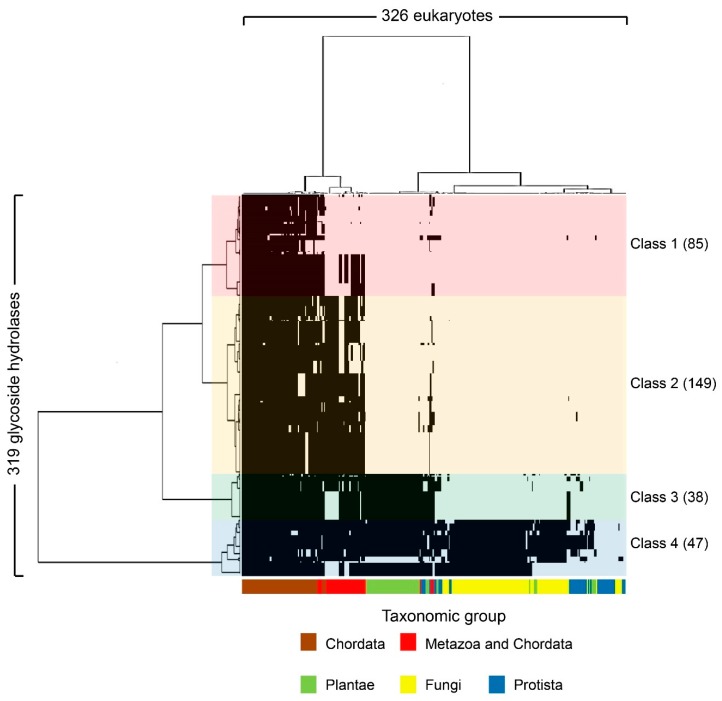
Phylogenetic profiling of human glycoside hydrolases. The *X*-axis shows 326 organisms (Table S3) that underwent genome sequencing, and the *Y*-axis shows the 319 human glycoside hydrolases (Table S2). Based on the phylogenetic tree, human glycoside hydrolases were classified into four characteristic clusters, defined as Classes 1–4, which included 85, 149, 38 and 47 human glycoside hydrolases, respectively. The black regions indicate the presence of human glycoside hydrolase orthologs in specific groups of organisms, shown in different colours on the *X*-axis. In Class 1, chordates or metazoans are included. In the metazoans in Class 1, some metazoan animals such as some fly species were excluded.

The 319 human glycoside hydrolases in the dataset were classified into four clusters by phylogenetic profiling (Figure 1, Tables S3 and S4) and cluster analysis. The four clusters included enzymes with orthologs primarily conserved in chordates or metazoans (Class 1), metazoans (Class 2), metazoans and plants (Class 3) and eukaryotes (Class 4). The molluscs *Octopus bimaculoides*, *Crassostrea gigas*, *Lottia gigantea* and cnidarian *Nematostella vectensis* were classified in the same cluster as the Chordata. Additionally, two deuterostome taxa, i.e., Choanoflagellatea and *Dictyostelium*, showed greater conservation relative to all fungi (Figure 1).

### 2.3. Functions of Human Glycoside Hydrolases Differ Among Classes

**Figure 2:**
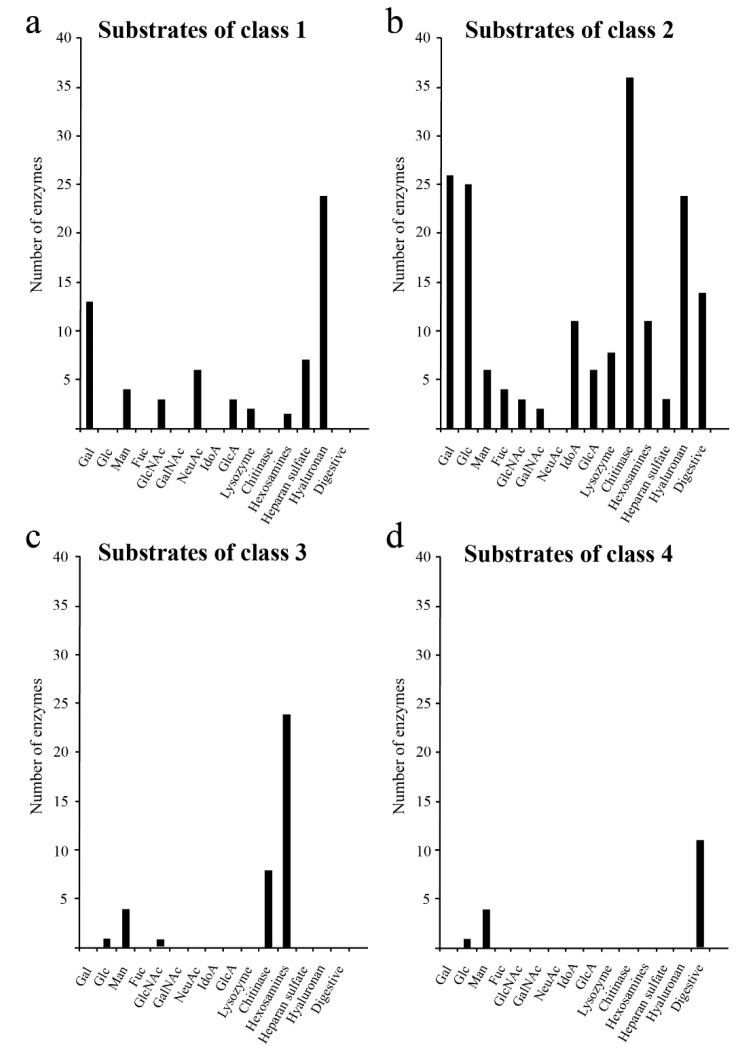
Degradation substrates of human glycoside hydrolases from each class. The X- and Y-axes show the degradation substrates and number of human glycoside hydrolases, respectively. Degradation substrates are shown for Class 1 (**a**), Class 2 (**b**), Class 3 (**c**) and Class 4 (**d**).

Next, we characterised the types of glycans degraded by each class of glycoside hydrolases. The results showed that Classes 1 and 2 (Figure 2a,b) contained glycoside hydrolases such as hyaluronidase, lysozyme and chitinase which degraded glycosaminoglycans, and glycoside hydrolases such as glucosyl ceramidase and sialidase that degraded glycolipids. Class 4 contained glycoside hydrolases that only degraded *N*-linked glycans (Figure 2d). Our analysis showed that glycoside hydrolases degrading galactose and *N*-acetylgalactosamine, which are commonly found in *O*-linked glycans, were unevenly distributed in Classes 1 and 2 (Figure 2). These results suggest that *O*-linked glycans were obtained after acquisition of *N*-linked glycans in the evolution of glycosyl hydrolases (GHs) as shown in the analysis of glycosyltransferases (GTs) [3].

### 2.4. Comparison of Decomposition Substrates Among Classes of Glycoside Hydrolases

**Figure 3:**
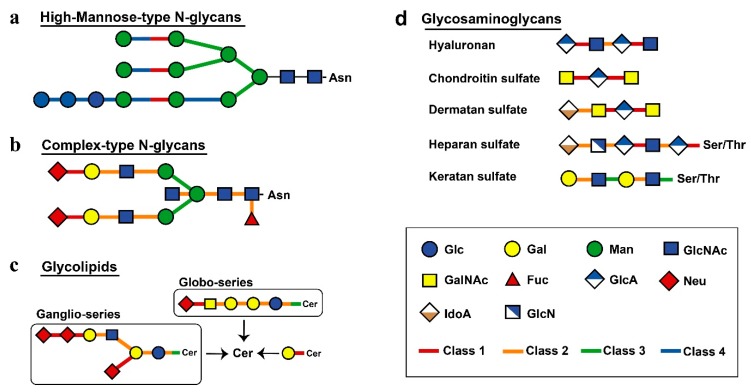
Mapping of evolutionary information to metabolic pathways. Different classes are shown by different colours, as indicated. The links connecting human glycoside hydrolase classes and degradation substrates show bonds degraded by each class of enzymes. The single glycan is shown based on the Consortium for Functional Glycomics symbol. The figure shows high-mannose-type *N*-glycans (**a)**, complex-type *N*-glycans (**b)**, glycolipids (**c)** and glycosaminoglycans (**d)**.

Next, we investigated other differences among the classes of sugar hydrolases. Substrates and products of human glycoside hydrolases were referenced according to the Kyoto Encyclopaedia of Genes and Genomes (KEGG) database [21], and the relationships between the degradation of glycan structures and glycoside hydrolases were mapped (Figure 3). Human glycoside hydrolases of the high-mannose-type *N*-linked glycans, particularly those with processing function, were widely conserved in eukaryotes (Figure 3a). Glycoside hydrolases classified into Class 2 or 3 were involved in the degradation of complex *N*-linked glycans. This result suggests changes in substrates from complex glycans to functional substances in human glycoside hydrolases that originated from multicellular organisms (Figure 3b). Glycans of *N*-linked glycoproteins and glycolipids were degraded by specific glycoside hydrolases at the nonreducing end (Figure 3c). Many glycoside hydrolases had exo-type functions allowing for the decomposition of monosaccharides at nonreducing ends. In contrast, glycoside hydrolases were classified into Class 1 had endo-type functions and acted to decompose the interior region of carbohydrate chains. An endo-type glycoside hydrolase was shown to enhance the efficiency of endoplasmic reticulum-associated degradation (ERAD) of folding-deficient proteins in the protein quality control process [22]. These findings suggest that the acquisition of a mechanism involved in alleviating endoplasmic reticulum stress contributed to chordate evolution. The structure of human glycosaminoglycans was largely degraded by glycoside hydrolases obtained from chordates, except keratan sulphate, which was decomposed by glycoside hydrolases from Classes 2 and 3 (Figure 3d).

### 2.5. Identification of Glycoside Hydrolases Important for the Evolution to Mammals

**Figure 4:**
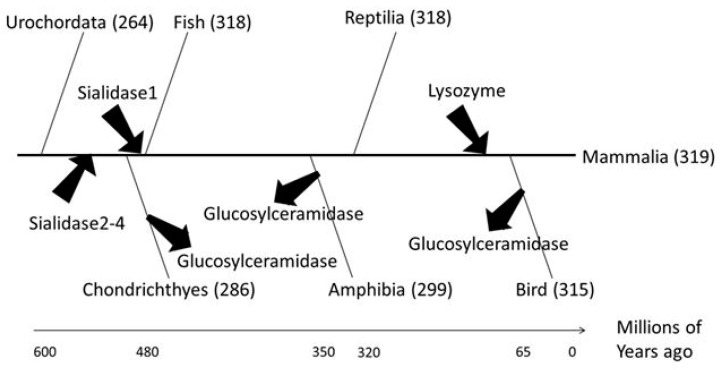
Evolution of human glycoside hydrolases. The time divergence analysis refers to Rowe’s tree of life [23]. The *X*-axis shows the time of evolution, and arrows indicate the acquisition or loss of human glycoside hydrolases.

Molecular phylogenetic analysis was conducted to investigate how human glycoside hydrolases evolved in the process of evolution from chordates to mammals (Figure 4). Sialidase, which is involved in neuronal and muscle differentiation, and lysozyme, which plays an important role in mammalian embryos, were acquired before the emergence of cartilaginous fish and of the common ancestor of birds and mammals, respectively. Glucosylceramidase, a Class 2 glycolipid-metabolising enzyme, was conserved in most Chordata but was lost during evolution in some chordates including *Gallus* spp. and *Xenopus laevis*. Accordingly, we hypothesised that sialidase, lysozyme and glucosylceramidase were necessary for the evolution to mammals.

### 2.6. Evolution of Glycosyltransferases and Glycoside Hydrolases

**Figure 5:**
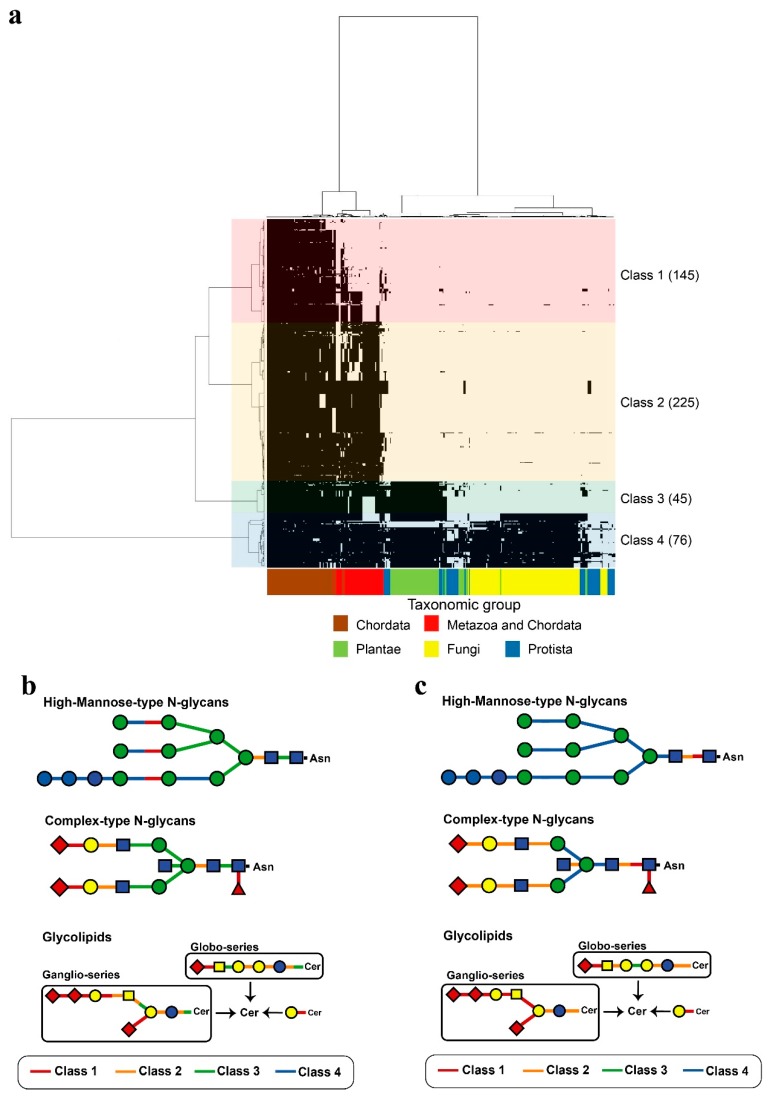
Phylogenetic profiling of human glycoside hydrolases and human glycosyltransferases. In (**a**), the *X*-axis indicates 326 organisms that underwent genome sequencing (Table S3), and the *Y*-axis indicates 319 human glycoside hydrolases and 172 glycosyltransferases (Tables S5 and S6). Based on the phylogenetic tree, the enzymes were classified into four characteristic clusters, defined as Classes 1–4, which included 145, 225, 45 and 76 enzymes, respectively. Classes are indicated by different colours. Links between human glycosyltransferases and glycoside hydrolases classes (**a**) and degradation (**b**) or synthesis (**c**) substrates are shown. The single glycan is shown based on the Consortium for Functional Glycomics symbol. Addition or removal of Neu (magenta triangle) occurs during sialic acid modification.

To compare the acquisition processes of human glycosyltransferases and human glycoside hydrolases (Table S5), phylogenetic profiling analysis of human sugar hydrolases and human glycosyltransferases was performed (Figure 5a, Table S6). The results showed that human glycosyltransferases and human glycoside hydrolases were classified into four characteristic clusters, defined as classes 1–4, based on their similarity to enzymes conserved in chordates or metazoans (Class 1), metazoans (Class 2), metazoans and plants (Class 3) and eukaryotes (Class 4). In this analysis, *Strongylocentrotus* were classified together with Class 1, whereas enzymes of the chordates *Ciona intestinalis* and *Branchiostoma floridae* were classified together with Class 2. However, degradation enzymes for the core structure of *N*-linked glycans had a lower degree of conservation in other organisms than that of human glycosyltransferases. Additionally, β-1,4-mannosyl-glycoprotein 4-β-*N*-acetylglucosaminyltransferase (MGAT3), a human glycosyltransferase of a bisecting GlcNAc, was found to be conserved in the complex type (*N*-glycan). Because few studies have evaluated human glycoside hydrolases, it was difficult to map *O*-glycans to a metabolic pathway; however, this *O*-glycan hydrolase was classified in the same cluster as a human glycosyltransferase. Additionally, when we focused on sialic acid modifications, which were shown to be required for protein stabilization and neuronal differentiation, the human sialic acid glycoside hydrolase and human sialic acid glycosyltransferase were found to have be acquired in the same period during evolution (Figure 5b,c).

### 2.7. Intrinsically Disordered Regions of Glycoside Hydrolases

**Figure 6:**
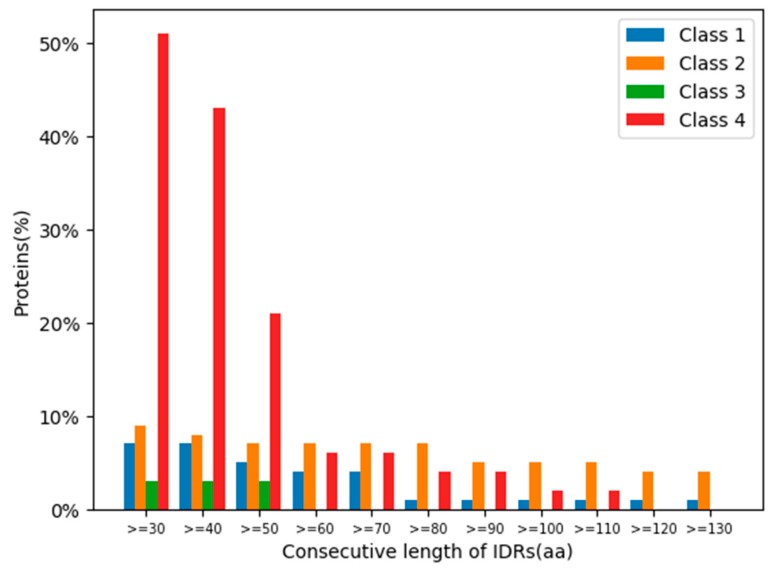
Percentages of glycoside hydrolases with specific consecutive lengths of intrinsically disordered regions (IDRs) in each class. Classes are indicated by different colours.

**Figure 7:**
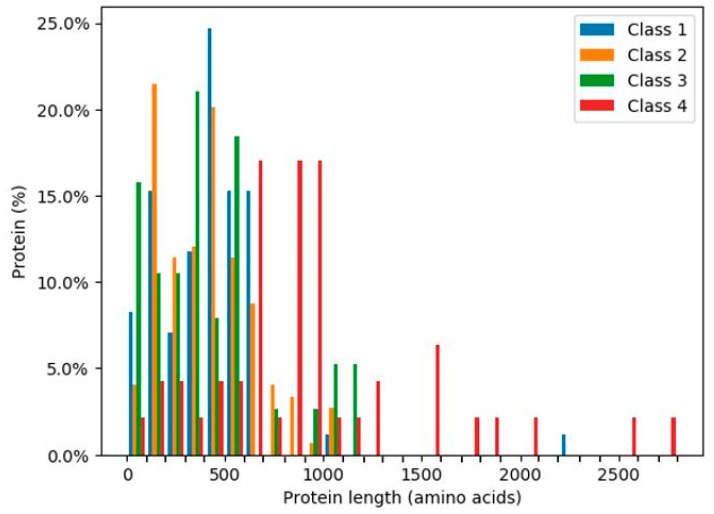
Percentages of glycoside hydrolases with the indicated lengths in each class. Classes are indicated by different colours.

The ratios of the lengths of intrinsically disordered regions (IDRs) to the total amino acid protein sequences [24] were analysed for the 319 human glycoside hydrolases (Figure 6 and Figure 7). The presence of continuous stretches of IDRs was predominant within Class 4, which showed significantly higher ratios than those in the other classes (Figure 6). More than 50% of Class 4 members had a continuous stretch of IDR of more than or equal to 30 amino acids, whereas only 10% or less members of the other classes had this continuous stretch of IDR. These results are consistent with the distribution of protein lengths, which were commonly longer among Class 4 members than among those from the other classes (Figure 7).

## 3. Discussion

In this study, we performed phylogenetic analysis of human glycoside hydrolases to evaluate the evolution of glycan-mediated biological systems. We found that 319 human glycoside hydrolases were classified into four clusters, including enzymes with orthologs in chordates, metazoans, metazoans and plants and eukaryotes. We also compared the dataset in this study to enzymes annotated by GO and found that 78.7% enzymes overlapped. Thus, most enzymes in the dataset of this study have already been annotated by using GO. Based on these findings, we propose that the acquisition of each human-type glycoside hydrolase gene was associated with the development of an intracellular protein-producing system and extracellular glycan-dependent biological interactions, as well as with the development and diversification of neuronal and neuromuscular functions. Consistent with data from a previous study showing that *N*-linked glycosyltransferases were widely conserved from the ancestral species of eukaryotes [3], the acquisition of high-mannose-type *N*-glycan-degrading enzymes occurred from ancestral species of eukaryotes. Among these enzymes, endo-β-*N*-acetylglucosaminidase and α-mannosidase 2C1 are localised in the endoplasmic reticulum, with the ERAD machinery facilitating accurate quality control of glycosylated proteins [25]. Similarly, the acquisition of high-mannose-type *N*-glycan-degrading enzymes was closely correlated with lectin-mediated glycoprotein folding [26]. Thus, precise regulation of *N*-glycan synthesis and degradation may play a central role in ensuring the integrity of *N*-glycan-mediated biological processes in eukaryotes. Our results showed that human-type *N*-glycan-degrading enzymes and the intracellular ERAD-related quality control of the protein-producing system were conserved throughout eukaryotes.

During the evolution of metazoans, polysaccharide-degrading enzymes such as lysozyme and chitinase, glycosaminoglycan-degrading enzymes and hyaluronidase were acquired. These molecules are essential in the defence against bacterial infections, as well as for fertilisation and ECM remodelling [27,28,29], therefore the acquisition of these degrading enzymes may play important roles in regulating glycan-mediated biological functions. Among the glycosaminoglycan-degrading enzymes, keratan sulphate-processing enzymes are involved in many biological processes, whereas other degrading enzymes such as hyaluronidases, chondroitinases, heparitinases and dermatan sulphate-degrading enzymes are mainly involved in neuronal functions [30]. Thus, during the evolutionary development of neuronal tissues, regulation of *O*-glycan modifications by *O*-glycan-degrading hydrolases may have played important roles in both plasma membrane-mediated and ECM-dependent biological functions. In terms of glycan degradation of *N*-glycans and glycolipids, ancestrally acquired human glycoside hydrolases can show degradation activity for the nonreducing end, whereas the sialic acid-degrading enzyme sialidase is essential for degrading the reducing end. Thus, complex-type *N*-glycans and glycolipids may have evolved by the addition of new sugars at the nonreducing end of ancestrally acquired glycans in multicellular organisms.

During evolution to chordates, an endo-α-mannosidase, MANEMA, was acquired. As described above, most exo-type mannosidases were acquired from ancestral eukaryotes, and the acquisition of the endo-type mannosidase MANEMA conferred organisms with the ability to efficiently degrade misfolded proteins. During evolution to chordates, genomic gains of sialidase genes occurred twice before the ancestral chordates evolved into teleosts. Sialic acid-mediated modification of proteins is essential for muscle, neuronal and lysosomal functions [30], therefore the acquisition of sialidases may have been essential for the development of neuronal and neuromuscular structures, and lysosome-mediated protein degradation systems during evolution to chordates.

**Figure 8:**
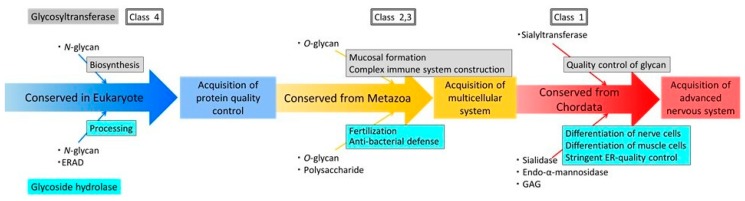
Schematic representation of a model for acquisition processes of human glycosyltransferases and human glycoside hydrolases using the results of a previous study [3] and this study. Blue arrows indicate enzymes that are widely conserved in eukaryotes; yellow arrows indicate enzymes that are conserved in metazoans; and red arrows indicate enzymes acquired from chordates.

In Rowe’s phylogenetic tree (Figure 8), sialidases and lysozyme were acquired during the evolution of mammals. Sialidases regulate higher cerebral functions, therefore the acquisition of these enzymes may have yielded more highly organised neuronal and muscular structures, facilitating the evolution of neuromuscular development. Similarly, the acquisition of lysozyme by ancestral species of mammals may have facilitated the development of the viviparous system in these organisms.

Although glucosylceramidase genes are conserved both in ancestral chordates and mammals, these genes disappeared during the diversification of Chondrichthyes, amphibians and birds. These results indicate that glucosylceramidases are essential enzymes regulating the mammalian-specific functions of glycolipids. Alternatively, glucosylceramidases may not have been essential but were continuously maintained during the evolution of mammals. Glucosylceramidases are highly regulated in higher vertebrates, therefore glucosylceramidase activity may have been essential for nervous system development in mammals. Further studies are required to confirm these hypotheses.

Most high-mannose-type human *N*-linked glycosyltransferases and *N*-glycoside hydrolases co-evolved in eukaryotes, therefore high-mannose-type human *N*-glycan-dependent ERAD is thought to be essential for the precise regulation of *N*-glycan-mediated biological processes. Similarly, both glycosyltransferases and glycoside hydrolases for glycolipids and *O*-glycans were acquired at nearly the same time and co-evolved together. Thus, the development and diversity of glycolipid- and *O*-glycan-mediated biological systems were likely essential for multiple functions, including formation of the mucous membrane system and highly organised immune system, in the evolution to metazoans and vertebrates. Complex-type bisecting GlcNAcs inhibit elongation of the β-1,6-GlcNAc branch at the nonreducing end of the core mannose of an *N*-glycan to stabilise the structure of the glycan, therefore we focused on the timing of the acquisition of bisecting GlcNAc hydrolase and transferase. In contrast to bisecting GlcNAc hydrolases, which were acquired from more distant ancestral species, the complex-type bisecting GlcNAc transferase MGAT3 was acquired later and is conserved in most metazoans. Complex-type bisecting GlcNAcs stabilise various biological functions including the E-cadherin-dependent cell adhesion system, therefore the acquisition of bisecting GlcNAc elongation enzymes may have been involved in the evolution of metazoans [31].

However, the best approach to the direct evolution of these glycoside hydrolases remains unclear. Previously, we suggested that the evolutionary origin and functional acquisition of proteins are closely related to their IDRs [24]. Our results showed that the most conserved class also contained the greatest number of consecutive stretches of IDRs. Additionally, Class 4 proteins commonly contain *N*-glycan-degrading enzymes and intracellular ERAD-related quality control proteins, such as ER degradation-enhancing mannosidase-like proteins (EDEMs), which are ER-resident members of the glycoside hydrolase 47 family, recruiting terminally misfolded polypeptides present in the ER lumen to the downstream ERAD pathway [23,32]. In this study, all EDEMs, including EDEM1–3, were predicted to have disordered regions. The presence of disordered regions at the N-terminus of EDEM1 has been reported previously based on modelling and prediction studies. These regions have been shown to be important for recognising glycosylated and non-glycosylated misfolded proteins, even when the carbohydrate-binding domain is highly impaired [23]. Long consecutively disordered residues (>30) may function as entropic chains or can be involved in interactions using combinations of recognition motifs or domains [33]. We previously reported that residues within disordered regions that function as entropic chains evolve quickly, whereas those involved in protein–protein interactions tend to be constrained [13,14]. Thus, it may be relevant for some ancient glycoside hydrolases to harbour long stretches of disordered regions because the conformational plasticity of these regions enables the recognition of or binding to multiple partners, which is beneficial for identifying misfolded proteins.

Several mechanisms may shape the evolution of GH. Despite gene duplication, acquisition of genes may occur through other processes. Some genes may be acquired de novo from a stretch of non-coding DNA. The acquisition of this gene may coincide with environmental conditions such as codfish antifreeze glycoprotein genes that have evolved de novo from non-coding DNA in the cooling time of its habitat 13–18 million years ago [34]. Another possible mechanism is horizontal gene transfer which involves the movement of transposable elements between different species; this mechanism is well-known in prokaryotes and unicellular eukaryotes and remains controversial and less established in higher organisms [34]. However, several studies have exemplified this case clearly in a complex organism such as GH genes that are found nearly exclusively and to the largest extent in western corn rootworm (*Diabrotica virgifera virgifera*) among insects and the presence of Bovine-B (BovB) retrotransposons in mammals [35,36]. In contrast, by utilizing symbiotic relationships such as gastrointestinal tract and microbiome, the acquisition of new genes or GH may not necessary to gain a function. In this case, some bacteria in the human gastrointestinal tract utilize their GH to cleave glycans that humans are unable to process; for instance, *Bifidobacteria longum* biovar *infantis* process oligosaccharides in milk that are not digestible by human infants [37]. The acquisition of GH by horizontal gene transfer from the microbiome also appears possible, but requires further analysis.

## 4. Materials and Methods

### 4.1. Human Glycoside Hydrolase Dataset

The glycoside hydrolase sequence data were obtained from UniProt (release 2017_03) [17] using the following queries: “glycoside hydrolase” and “organism: human”. To confirm the annotation of each retrieved sequence as glycoside hydrolase, we extracted all UniProt IDs within the glycoside hydrolase category (EC3.2) from the CAZy [18] and BRENDA [19] databases and confirmed the presence of the UniProt ID for each retrieved sequence in the CAZy [18] and BRENDA [19] databases; unannotated sequences in any of these databases were removed. This was an alternative method used to obtain more data on human glycoside hydrolase sequences than would be obtained by using GO [38] and InterPro [20] using InterPro entry glycoside hydrolase superfamily (IPR017853), and was the easiest way to obtain human glycoside hydrolases with UniProt IDs in CAZy and BRENDA. In addition, we verified our data with glycoside hydrolases obtained using GO, 553 glycoside hydrolases that have been annotated as GO: 0016798, Taxon: *Homo sapiens* were isolated and compared to the dataset. Further, to analyse the evolution of glycoside hydrolases, we categorised these enzymes based on their substrates and products into four categories including *O*-glycans (mucins), *N*-glycans (high-mannose type, complex type), glycolipids and glycosaminoglycans based on the metabolic map in the KEGG database [21].

### 4.2. Phylogenetic Profiling and Cluster Analyses

Phylogenetic profiles were generated for 326 genome-wide eukaryotic sequences using KEGG OC default parameters in the KEGG database and extracted human glycoside hydrolase data as queries. Human glycoside hydrolase conservation in eukaryotes was examined using a BLAST search (E-value: 10−3; NIH). A bit score of 1 was assigned if orthologs of the protein of interest were present in the other genome; otherwise, a bit score of 0 was assigned. Proteins with similar bit patterns were expected to have similar interactions and functions. Further, using the bit pattern as an input, cluster analysis of the 319 human GHs and 326 eukaryotes from KEGG OC were performed using Ward’s method [39] based on the Manhattan distance. Computational and cluster analyses were performed using Ruby and R programming languages.

### 4.3. Molecular Phylogenetic Analysis

A phylogenetic tree of glycoside hydrolases was manually constructed, and a model for the time divergence of chordates to mammals during evolution was presented as described by Rowe [40].

### 4.4. Protein IDR Analysis

Human glycoside hydrolases were classified based on structural order/disorder into three categories: structured proteins, proteins with structured domains and disordered regions and intrinsically disordered proteins (IDPs). Allocation into these categories was performed according to the proportion of short IDRs (functional regions) of 15 residues [41]. The structured proteins were defined as proteins without any IDRs; IDPs were defined as proteins with IDRs spanning throughout the entire sequence, and the last category included proteins made up of both IDRs and structured regions [33]. The structural order/disorder propensity of the dataset was predicted using IUPred2a with 0.5 as the cut-off between order and disorder [42]. A value of 0 indicated a strong propensity for being ordered, and that of 1 indicated a strong propensity for being disordered. Continuous stretches of IDRs were plotted at n ≥ 30, 40, …, 130, as a stretch of more than 30 residues was required for categorisation as a long disordered region, with potential functions in recognition or interactions [33,43].

### 4.5. Source Code

The source codes used for our experiments are available at https://github.com/ritsumei-infobio/phylogenetic_profiling.

## 5. Conclusions

In summary, we performed genome-wide phylogenetic profiling and cluster analysis of human glycoside hydrolase proteins. Our results suggest that the acquisition of human glycoside hydrolase genes was essential for the development of the intracellular ERAD system in eukaryotes and for glycan-dependent extracellular signalling in multicellular organisms. Analysis of human glycoside hydrolase genes using Rowe’s phylogenetic tree indicated that the modulation of glycan-dependent biological functions by sialidases and lysozyme and that the divergence of glucosylceramidases occurred during chordate evolution (Figure 8).

## Supplementary Materials

Supplementary Materials can be found at https://www.mdpi.com/1422-0067/20/24/6290/s1.

## Abbreviations

UniProt	The Universal Protein Resource
CAZy	Carbohydrate-Active Enzymes
KEGG	Kyoto Encyclopedia of Genes and Genomes
O-linked	Oxygen-linked-type
ECM	Extracellular matrix
N-linked	Nitrogen-linked-type
GO	Gene Ontology
GHs	Glycosyl hydrolases
ERAD	Endoplasmic reticulum-associated degradation
MGAT3	β-1,4-mannosyl-glycoprotein 4-β-N-acetylglucosaminyltransferase
GlcNAc	N-acetylglucosamine
N-glycan	N-linked glycan
O-glycan	O-linked glycan
IDRs	Intrinsically disordered regions
EDEMs	ER degradation-enhancing mannosidase-like proteins
BovB	Bovine-B
IDPs	Intrinsically disordered proteins
