# Supplementary material for: Genome-Wide Analysis of Whole Human Glycoside Hydrolases by Data-Driven Analysis in Silico
Source: Int J Mol Sci. 2019 Dec 13;20(24):6290. doi: 10.3390/ijms20246290 (PMC6940844; doi:10.3390/ijms20246290)
Supplement: Supplementary file 1 [file ijms-20-06290-s001.zip › ijms-660336 SI/ijms-660336-SI-Author Proofreading .docx]

Supplementary Materials

**Supplementary Data 1.** Glycoside hydrolase dataset used in this study from Uniprot and its overlapping status with dataset in GO. The tables show the comparison of dataset retrieved from Uniprot and GO.

**Supplementary Data 2**. Accession number of the glycoside hydrolase dataset used in this study (UniProt December, 2018). The table includes the data information of entry name, protein name, and status in the UniProt database, the result of clustering based on the information of the phylogenetic profiling, and the ordered-disordered structure information including percentage of ordered-disordered residue, the absence and presence of continuous stretch of disordered peptide, structure category and the longest continuous stretch of disordered region (Figure 1).

**Supplementary Data 3.** Organisms list (KEGG June, 2016). The table shows the list of organisms used for generating phylogenetic profiling.

**Supplementary Data 4.** The result of phylogenetic profiling of human glycoside hydrolases. The column headers indicate the KEGG organism ID (Table S3), and the row headers indicate the UniProt ID of human glycoside hydrolases (Table S2). The 1 and 0 values indicate the presence and absence of organism ortholog glycoside hydrolase to human, respectively.

**Supplementary Data 5.** Accession number of the glycoside hydrolase and glycosyltransferase dataset (UniProt December, 2018). The table shows the information of human glycosyltransferase used in the previous study^3^ and human glycoside hydrolase used in this study. The table includes the data information of entry name, protein name and status in the UniProt database and the result of clustering based on the information of the phylogenetic profiling (Figure 5a).

**Supplementary Data 6.** The result of phylogenetic profiling of human glycoside hydrolases and glycosyltransferase. The column headers indicate the KEGG organism ID (Table S3), and the row headers indicate UniProt ID of human glycoside hydrolases and glycosyltransferase (Table S5). The 1 and 0 values indicate the presence and absence of organism ortholog glycoside hydrolase to human, respectively.
